# Application of spectral image processing with different dimensions combined with large-screen visualization in the identification of boletes species

**DOI:** 10.3389/fmicb.2022.1036527

**Published:** 2023-01-12

**Authors:** Jie-Qing Li, Yuan-Zhong Wang, Hong-Gao Liu

**Affiliations:** ^1^College of Agronomy and Biotechnology, Yunnan Agricultural University, Kunming, China; ^2^Medicinal Plants Research Institute, Yunnan Academy of Agricultural Sciences, Kunming, China; ^3^Zhaotong University, Zhaotong, China

**Keywords:** boletes species, 2DCOS images, 3DCOS images, Alexnet, Resnet, large-screen visualization

## Abstract

Boletes are favored by consumers because of their unique flavor, rich nutrition and delicious taste. However, the different nutritional values of each species lead to obvious price differences, so shoddy products appear on the market, which affects food safety. The aim of this study was to find a rapid and effective method for boletes species identification. In this paper, 1,707 samples of eight boletes species were selected as the research objects. The original Mid-Infrared (MIR) spectroscopy data were adopted for support vector machine (SVM) modeling. The 11,949 spectral images belong to seven data sets such as two-dimensional correlation spectroscopy (2DCOS) and three-dimensional correlation spectroscopy (3DCOS) were used to carry out Alexnet and Residual network (Resnet) modeling, thus we established 15 models for the identification of boletes species. The results show that the SVM method needs to process complex feature data, the time cost is more than 11 times of other models, and the accuracy is not high enough, so it is not recommended to be used in data processing with large sample size. From the perspective of datasets, synchronous 2DCOS and synchronous 3DCOS have the best modeling results, while one-dimensional (1D) MIR Spectrum dataset has the worst modeling results. After comprehensive analysis, the modeling effect of Resnet on the synchronous 2DCOS dataset is the best. Moreover, we use large-screen visualization technology to visually display the sample information of this research and obtain their distribution rules in terms of species and geographical location. This research shows that deep learning combined with 2DCOS and 3DCOS spectral images can effectively and accurately identify boletes species, which provides a reference for the identification of other fields, such as food and Chinese herbal medicine.

## 1. Introduction

Wild edible mushrooms are rich in protein, vitamins and a variety of mineral elements. Regular consumption can enhance immunity and promote metabolism. In addition, it still has medicinal value, can effectively prevent and treat tumors, edema and other diseases (Li et al., 2008; Cheung, 2010; Yao et al., 2018). Recently, it has been reported in the literature that mushrooms, including *Ganoderma lucidum*, should be considered as functional foods. It also pointed out that *Ganoderma lucidum* can treat coronavirus disease of 2019 (COVID-19; El Sheikha, 2022). Another study suggests that mushrooms can be used as bioinsecticidal agents (El Sheikha, 2021). China is the world’s largest producer of edible mushrooms and truffles (El Sheikha and Hu, 2018). Moreover, China is the main producer and exporter of edible mushrooms, accounting for more than 70% of the world’s annual output, with a total output of approximately 36 million tons, and export trade is up to more than 130 countries (Zhang et al., 2020; Yan et al., 2021). Bolete is a world-famous high-quality edible fungus. China is rich in natural resources and has great potential for its development and utilization. Yunnan has a unique climate and diverse vegetation, which provides a good environment for the growth of wild boletes, so it is one of the largest boletes production areas in the world (Yang et al., 2016, 2017). 882 species of wild edible mushrooms are known to be distributed in Yunnan, accounting for 44.1% of the 2000 species in the world and 91.3% of the 966 species in China. In 2021, Yunnan province produced 280,400 tons of wild edible mushrooms, among which the boletes from Chuxiong are nationally famous^1^. Nevertheless, the price of boletes varies greatly depending on species, and some species of boletes may cause poisoning. At present, there are illegal traders in the market who use fake as real and use shoddy as good, which damages the interests of consumers and affects food safety at the same time (El Sheikha, 2018; Yan et al., 2022a). Therefore, rapid and accurate identification of bolete species is urgently required.

Mass spectrometry, chromatography, spectroscopy and other methods are often used to evaluate the quality of wild edible mushrooms. Malheiro et al. used headspace solid phase microextraction (HS SPME) and gas chromatography/ion trap mass spectrometry (GC/IT MS) to analyze the volatilities of edible mushrooms, and used stoichiometric methods to identify six species of edible mushrooms. The results showed that volatile substances could be used as an important classification basis (Malheiro et al., 2013). Marekov et al. (2012) used gas chromatography-mass spectrometer (GC–MS) to determine 31 fatty acid components of 15 edible mushrooms belonging to 9 genera and 5 families in Bulgaria. Stoichiometric methods were used to analyze the differences in fatty acid components and classify the species. Mohač ek-gro šev et al. used Fourier transform infrared spectroscopy (FTIR) to make spectral analysis of the spores and fruiting bodies of more than 70 species of wild edible mushrooms belonging to 37 genera. The results showed that vibration spectroscopy could characterize the information of the content of polysaccharides in fungi, and the vibration spectra of spores of different species of the same genus and some of the fruiting bodies were very similar. The chemical composition of cap and stipe of the same fruiting body was quite different (Mohacek-Grosev et al., 2001). Each of these reported identification methods has advantages and disadvantages. Among them, morphological taxonomy identification technology is easily affected by subjective factors, and the identification accuracy is low. Mass spectrometry and chromatography are expensive and require expertise and technology (Mohacek-Grosev et al., 2001; Li et al., 2011, 2013). FTIR is an efficient and nondestructive discrimination technique, but its accuracy is not high because of spectral overlap.

In recent years, 2DCOS technology has been used to identify adulterants in food and herbal medicines. Walkowiak et al. (2019) successfully detected ginkgo biloba adulterants in dietary supplements using 2DCOS, and initially established an effective evaluation method for screening ginkgo biloba adulterants. Chen et al. (2018) used i2DCOS to visually identify adulterated medicinal materials. The results showed that i2DCOS technology has potential development in the visual identification and quality control of traditional Chinese medicine and other complex mixtures. Ma et al. (2016) used 2DCOS to accurately identify seven species of bolete mushrooms of the same genus, and applied the 2DCOS method to the identification of edible mushrooms. Nevertheless, these reports all use traditional machine learning methods that require complex data processing, high time costs, and accuracy needs to be improved. Recently, Dong et al. (2021b) used deep learning combined with 2DCOS and i2DCOS to identify the origin of *Boletus Edulis*, Yan et al. (2022b) used Resnet combined with 2DCOS to identify bolete species, Yue et al. (2021) used the same method to identify the part and region of the medicinal plant. However, the deep learning methods in these reports are single, only Resnet is employed, and there is a lack of comparison with other deep learning models. Moreover, only 2DCOS data were obtained, so fewer datasets can be used.

In this paper, the generation method of the 3DCOS dataset is proposed for the first time, which is expanded from single 2DCOS images to 1D MIR spectral images, 2DCOS images and 3DCOS images. Among them, 2DCOS and 3DCOS datasets include synchronous spectrum, asynchronous spectrum and integrative spectrum, which significantly increases the types of datasets and enables full verification of the model on each dataset. At the same time, we used two deep learning algorithms, Alexnet and Resnet, to process the acquired data sets one by one and compared them with the traditional SVM algorithm. Finally, the optimal method for identifying species of boletes was obtained. In addition, in order to analyze the species information and sample distribution of boletes more intuitively, we developed a large-screen of boletes data visualization using ECharts, HTML, CSS and JavaScript technology. We hope that the results of this study and the large-screen visualization technology can be extended to the field of food and traditional Chinese medicine identification.

## 2. Materials and methods

### 2.1. Sample collection and preparation

We collected 1,707 samples of boletes which belong to eight species from 46 sampling sites in 13 regions of Southwest China. All samples were identified by Professor Honggao Liu from Yunnan Agricultural University. The appearances of these 8 boletes species are shown in Supplementary Figure S1. The geographical information and other details of the sample are shown in Supplementary Table S1. In the laboratory, the sundries on the samples were removed, and they were cleaned with a SY3200-T type ultrasonic cleaning instrument. After that, the materials were dried to constant weight with a dryer at medium temperature. Finally, they were pulverized with a FW-100 high speed grinder, the power was sieved by an 80 μm mesh and stored for later use.

### 2.2. FT-MIR spectra acquisition

Precisely weigh 1.5 ± 0.2 mg powder of each sample and 150 ± 20 mg KBr powder in the ratio of 1: 100, put them into agate mortar, mix and grind them into fine powder. Then pour the fine powder into a pressed grinding tool to produce thin slices with uniform thickness. The sample was measured after preheating the Fourier transform infrared spectrometer for 30 min, and the sample was repeated twice to get the average spectrum. The interference of CO_2_ and H_2_O was deducted from the blank sample before scanning.

### 2.3. The spectral images acquisition

In this paper, 11,949 spectral images were obtained from 1707 boletes samples, including 1D MIR spectrum, 2DCOS (synchronous, asynchronous, integrative) and 3DCOS (synchronous, asynchronous, integrative). Among them, the theoretical basis of 2DCOS is as follows.

As shown in Equation 1, *t* represents the interval of perturbation, *m* represents the number of steps measured by the spectrum, the column vector *P* represents the dynamic spectral intensity at variable *v* (Yang et al., 2020).


(1) $$P\left(v\right)=\left[\begin{array}{c} \begin{array}{c} p\left(v,t_{1}\right)\\ p\left(v,t_{2}\right) \end{array}\\ .\\ .\\ .\\ p\left(v,t_{m}\right) \end{array}\right]$$

Then, the synchronous 2DCOS (*Φ*), asynchronous 2DCOS (*ψ*) and integrative 2DCOS (*I*) can be calculated as (Cheung, 2010; Chen et al., 2018; Dong et al., 2021b, 2022):


(2) $$\Phi \left(v_{1},v_{2}\right)=\frac{1}{m-1}P{\left(v_{1}\right)}^{T}\cdot P\left(v_{2}\right)$$

(3) $$\Psi \left(v_{1},v_{2}\right)=\frac{1}{m-1}P{\left(v_{1}\right)}^{T}\cdot N\cdot P\left(v_{2}\right)$$

$$\;I\left(v_{1},v_{2}\right)=\Phi \left(v_{1},v_{2}\right)\cdot \Psi \left(v_{1},v_{2}\right)$$

(4) $$=\frac{1}{{\left(m-1\right)}^{2}}\left[P{\left(v_{1}\right)}^{T}\cdot P\left(v_{2}\right)\right]\cdot \left[P{\left(v_{1}\right)}^{T}\cdot N\cdot P\left(v_{2}\right)\right]$$

where *N* is the Hilbert-Noda matrix, which is defined as:


(5) $$N_{jk}=\{\begin{array}{c} 0,j=k\\ \frac{1}{\pi \left(k-j\right)},j\neq k \end{array}$$

If there are *m* samples, then the 1D spectral data of all samples can be converted into an *m*×*n* data matrix, where *n* is the feature points contained in the spectrum. In this study, the matrix *P* (*m*×*n*) contains two spectra data (that is, *m = 2*), the average spectrum is the first and the *i*-th spectrum about each species of boletes is the second (Yang et al., 2013; Yang R. J. et al., 2014; Dong et al., 2021a).

**Figure 1:**
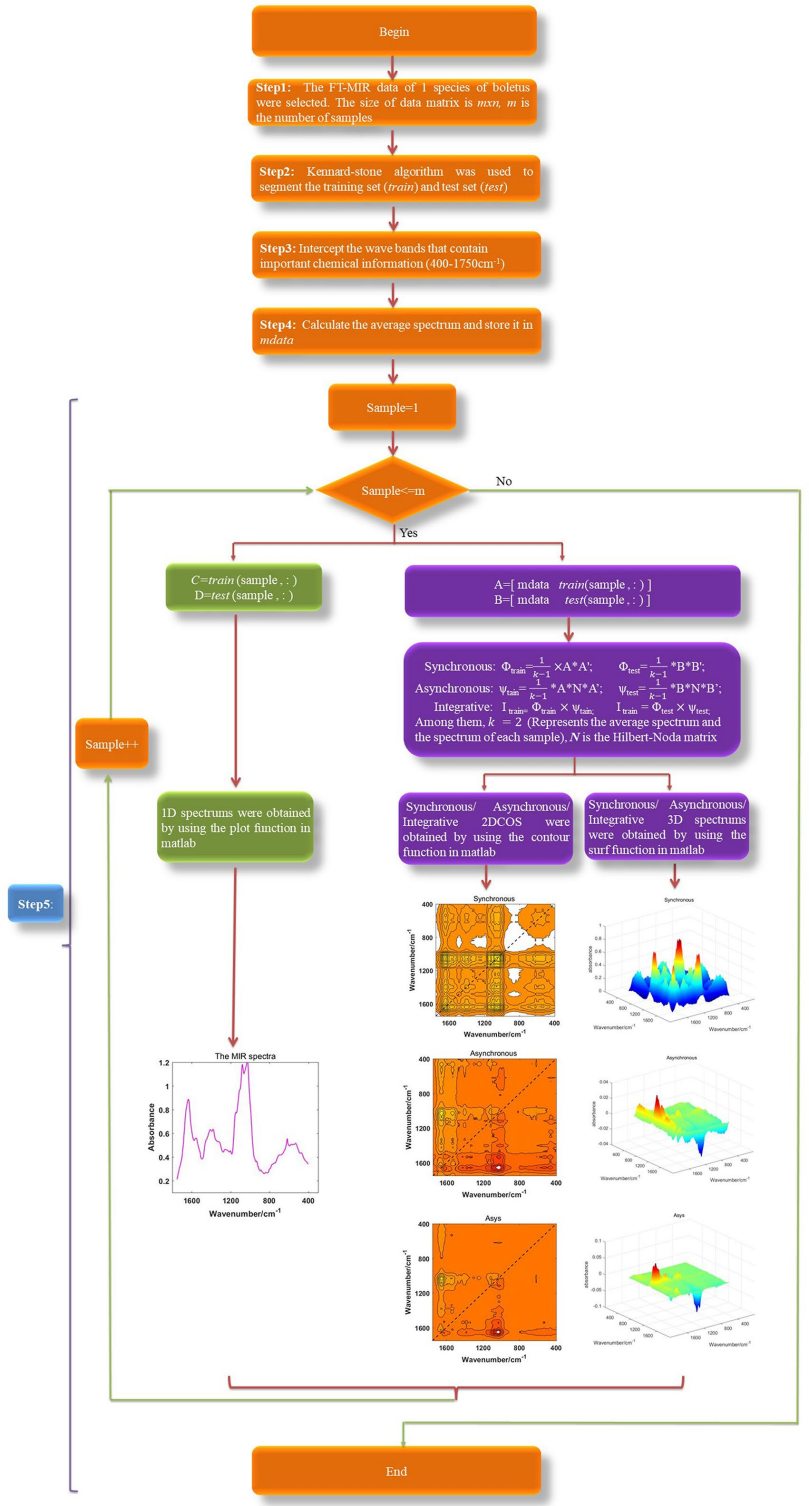
The spectral image generation processes, Asys: integrative.

The synchronous 3DCOS, asynchronous 3DCOS and integrative 3DCOS is generated in the same way as 2DCOS, but using 3D display. We intercept 1,750–400 cm^−1^ fingerprint regions to generate various spectral images to reduce the computational burden. To improve efficiency and avoid generating spectral images one by one like Origin and OMNIC software, we wrote a program based on Matlab2017 to generate and automatically save these spectral images in bulk. All the spectral image generation processes are shown in Figure 1. Firstly, all spectral data were obtained, and Kennard–Stone algorithm was used to divide the train set and test set. Then, various spectral images were obtained according to Equations 2–4 and different display methods. Finally, these images were separately saved to prepare for later model establishment.

### 2.4. Establishment of the identification model

#### 2.4.1. SVM model

SVM is a classification method based on the realization of the minimization of constitutive risk. With strong generalization ability and good robustness, it is widely used in image recognition, text classification, biotechnology, face recognition and other fields (Hu et al., 2022). Based on the limited sample information, SVM seeks the best between the complexity of the model and the learning ability, in order to obtain the best generalization ability. It shows unique advantages in solving small samples, nonlinear and high-dimensional pattern recognition (Li et al., 2022). The SVM model has two very vital parameters *c* and *g* where *c* is the penalty coefficient. If *c* is too large or too small, the generalization ability of the model will deteriorate. If *g* increases, the number of support vectors increases which will affect the speed of training and prediction. Therefore, the SVM model has a good effect on small samples, but is not suitable for large samples. In this paper, libsvm-3.21 toolbox was used to identify eight species of boletes.

#### 2.4.2. Alexnet model

Alexnet model was proposed by Alex in 2012 and won the championship of the 2012 image recognition competition, which makes CNN become the core algorithm model in image classification.

As is shown in Supplementary Figure S2, the input data of the first layer is the original 64×64 size image, which is convolved by 96 11×11 convolution kernels, and each convolution of the original image generates a new pixel, batch normalization is then performed. These pixel layers are processed by the Maxpooling operation. The scale of the pooling operation is 3×3, and the step size of the operation is 2, so the size of the pooled image is (64–3)/2 + 1 = 32 and the image of 32×32×96 is obtained. The input data of the second layer is the pixel layer of 32×32×96 after the first pooling, which is convolved by 256 convolution kernels of 5 ×5 and then batch normalized. After the pooling operation with the scale of 3×3 and the operation step of 2, 16×16 ×256 images are obtained. Then, it passes through three convolution layers with a convolution kernel of 3 × 3 and stride of 1, and the number of convolution kernels is 384,384,256, respectively. The third Maxpooling operation is also with the scale of 3×3 and the operation step of 2. Therefore, the size of the input data in the sixth layer is 8×8×256. The operation results are output through 4,096 neurons, and then these 4,096 neurons in the seventh layer are fully connected to output 4,096 data. Lastly, the trained value is output after being fully connected to 8 neurons in the eighth layer.

In order to run Alexnet model, we used an 8-core 16G ECS cloud server, selected tensorflow deep learning framework developed by Google, installed anoconda3 deep learning environment, and used matplotlib library to visualize output data. All spectral images were uniformly clipped to 64×64 before input to the model.

#### 2.4.3. Resnet model

ResNet was proposed by Microsoft Research’s Kaiming He in 2015, and it won first place in the ImageNet competition Classification task. It solves the problems of overfitting, weight attenuation and gradient disappearance caused by the deepening of the layer of CNN, and has excellent performance.

According to the Resnet theory, we first construct the identity residual block and the convolution residual block. When the dimension of input data is consistent with the dimension of output data, the identity residual block (identity block) is employed, while the convolution residual block (conv block) is used when the dimension of input data is inconsistent with that of output data. Then a 12 layers Resnet network display in Supplementary Figure S3 is established based on these two residual blocks. 1D, 2DCOS, and 3DCOS images were fed into the model as input data, and convolution, batch normalization, Relu nonlinear activation were performed first. Then three identity blocks and two conv blocks are used for processing. After that, global average pooling (GAP) and flatten were carried out. Finally, softmax was used to output the discrimination results.

Similarly, we used an 8-core 16G ECS cloud server, selected Amazon’s Mxnet deep learning framework, installed anoconda3 deep learning environment, and used matplotlib library to visualize output data. All spectral images were uniformly clipped to 64×64 before input to the model.

#### 2.4.4. Data set partition

In classical machine learning, the ratio of training set to test set is 7: 3 or 8: 2. In general, the larger the proportion of samples in the training set, the more data information it contains, and the better the model training results will be. In order to enhance the accuracy of SVM model, the ratio of 8: 2 was selected to divide the training set and the test set. For deep learning based on image processing, the generalization ability of its model is essential. In order to evaluate the generalization error of the model and make a choice through the test set, the training set and test set of Resnet and Alexnet models are divided into 7: 3. The specific division results are illustrated in Supplementary Table S2.

### 2.5. The identification strategy of boletes species

The identification strategy of eight boletes species is shown in Supplementary Figure S4. There are two modeling methods. One method is to directly use spectral data for SVM modeling, and the other method is to convert spectral data into images, then conduct deep learning modeling based on image processing. We convert the spectral dataset into seven image datasets including 1D, 2D (synchronous, asynchronous, integrative), and 3D (synchronous, asynchronous, integrative). Then, seven Resnet and seven Alexnet models were established respectively, and the final identification results were output. Therefore, a total of 15 recognition models based on traditional machine learning and deep learning are established in this paper.

### 2.6. The large-screen visualization

Data visualization large-screen is a means to transform boring, professional and unintuitive data content into interesting, simple and intuitive content with the help of visual language expression and convey it to large-screen viewers. With the support of the current new technology, data visualization is not only visible, but also communicative and interactive. The essence of this technique is the mapping of data space to graphics space (Yao, 2021).

In this paper, based on ECharts, HTML, CSS and JavaScript technology, a large-screen of boletes information visualization was developed to display the origin, sampling site, species and other information of boletes from various aspects. The layout and proportion of large-screen panels are shown in Supplementary Figure S5. The front-end editor uses Visual Studio Code. ECharts is a pure JavaScript diagram library, the bottom layer depends on the lightweight Canvas class library ZRender, based on BSD open source protocol, is a very excellent visual front-end framework. HTML is a markup language used to design web pages. The files written by the browser are interpreted and executed. On the HTML page, you can write program segments nested in scripting languages. CSS is a computer language used to represent the style of HTML files. It can not only statically decorate web pages, but also dynamically format the elements of web pages with various scripting languages. JavaScript is a scripting language embedded in HTML and used in the browser to add interactive behavior to the HTML page. It is directly embedded in the HTML page and interpreted by the browser to execute the code without precompilation.

## 3. Results and discussion

### 3.1. MIR spectral analysis

**Figure 2:**
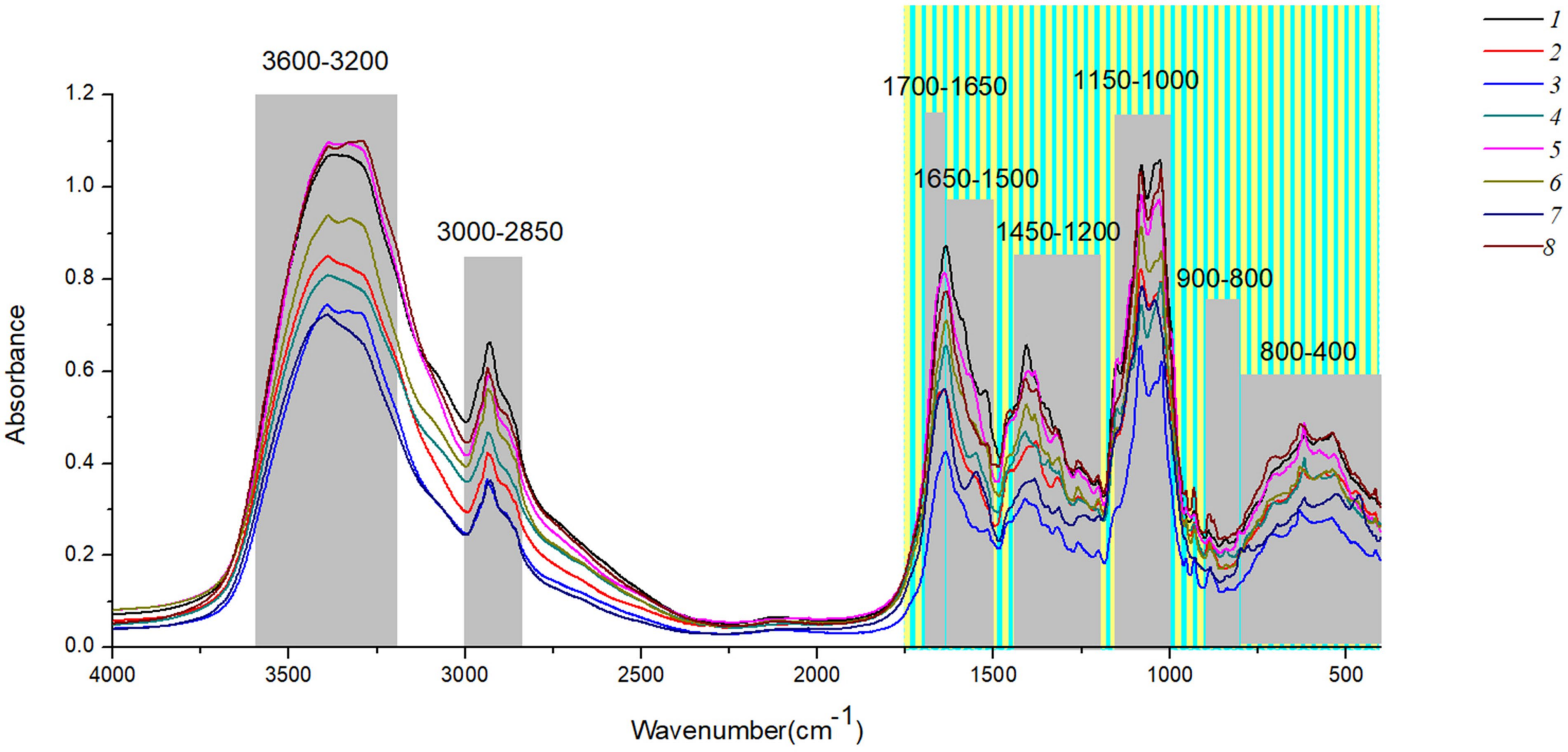
The original spectra of MIR of eight boletes species.

Figure 2 shows the original spectra of MIR of eight boletes species. The band 3,600–3,200 cm^−1^ is caused by O-H stretching, and the peak 3,342 cm^−1^ represents strong water interference (Hirri et al., 2016; Wang, 2020). The 3,000–2,850 cm^−1^ band is caused by the stretching of lipid methylene group and the pyranose ring, among which 2,928 cm^−1^ and 2,855 cm^−1^ are the absorption peaks of fatty acid components (Zhao et al., 2015; He, 2019). The 1700–1,000 cm^−1^ band contains the organic material, C-C stretching, C-O-H, C-H and CH_2_ bending (Mohacek-Grosev et al., 2001; Nie et al., 2007; Dong et al., 2021b). 1700–1,650 cm^−1^is mostly protein, 1,650–1,500 cm^−1^ mainly caused by amide І and amide П. The main components of 1,450–1,200 cm^−1^ are proteins, fatty acids, and polysaccharides (Chen et al., 2021; Dong et al., 2021b). In addition, the 1,000–1,200 cm^−1^ band is caused by carbohydrates, and the two peaks 1,032 cm^−1^ and 1,080 cm^−1^ mainly contain chitin (Yang T. W. et al., 2014; Zhang et al., 2018). Finally, the interval of 900–400 cm^−1^ can be effectively used for chemometric analysis, among which the band of 900–800 cm^−1^ mainly contains glucan and mannan (Qi et al., 2018; Chen et al., 2021). Supplementary Table S3 summarizes the Peak assignments on the FT-MIR spectra of boletes in different characteristic bands.

### 3.2. Spectral image analysis of three dimensions

**Figure 3:**
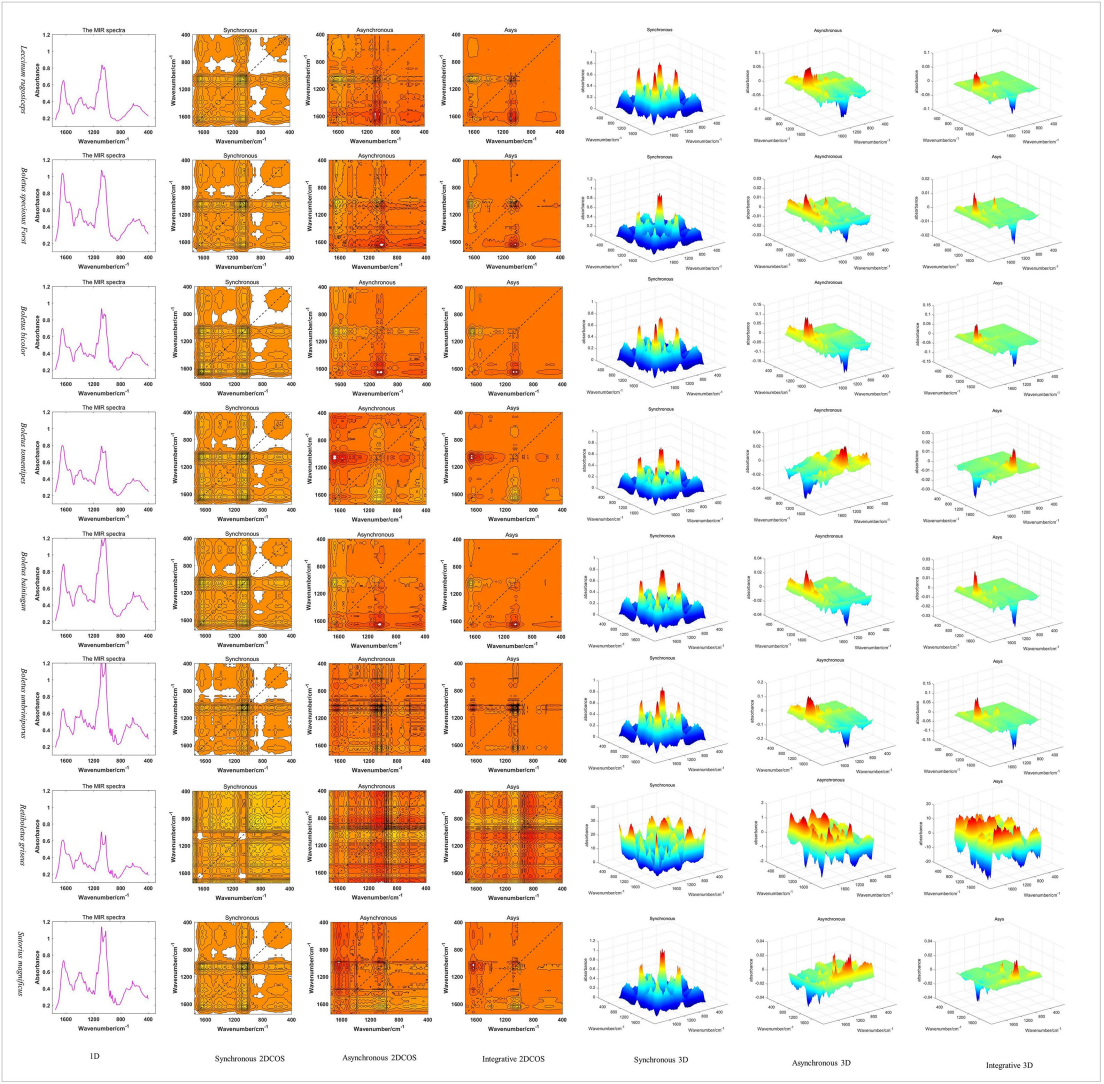
The spectral images of three dimensions about eight boletes species.

In this paper, Matlab2017 was used to generate 1D MIR spectral images in batches, and 2DCOS and 3DCOS images were generated according to relevant theories. The spectral images of three dimensions about eight boletes species are shown in Figure 3. It can be seen that the 1D spectra have obvious absorption peaks near wave numbers 3,300, 2,900, 1,640, 1,420, 1,070, 1,040, and 650 cm^−1^. However, the absorption peak is difficult to show due to spectral overlap, and the spectra are too similar to distinguish the eight boletes species. 2DCOS can show more information because the peak is expanded by the correlation operation. The synchronous 2DCOS has strong auto-peaks at 1650, 1080, 550 cm^−1^ and obvious cross-peaks at 1650, 1500, 1,080, and 550 cm^−1^. Asynchronous 2DCOS has no auto-peak, but it has strong cross-peaks at 1650, 1080 and 550 cm^−1^. The integrated 2DCOS only has two cross-peaks at 1650 and 1,080 cm^−1^, which contains relatively little information. 3DCOS is a 3D display of 2DCOS, and the size and number of peaks can be seen more intuitively from the 3DCOS. Among them, the synchronous 3DCOS has two large auto-peak at 1080 and 550 cm^−1^, a relatively small auto-peak at 1650 cm^−1^, and two large cross-peaks at 1080 and 1,500 cm^−1^. In addition, there are several smaller cross-peaks. Both asynchronous 3DCOS and integrated 3DCOS have two large cross peaks, one of which is positive and the other is negative. Therefore, from the distribution of peak values, synchronous 3DCOS can better identify boletes species. However, there are exceptions, such as *Retiboletus griseus*, which has many relatively large auto-peaks and cross-peaks.

### 3.3. Discrimination results of SVM model

In order to establish the SVM model, we selected 1,367 samples (80%) as the training set, and the remaining 340 samples (20%) as the test set. The Kennard–Stone algorithm was used to partition the dataset. Supplementary Figure S6A displays the optimal SVM parameters obtained by grid search method, among which the best *c* = 1.049×10^6^, the best *g* = 0.125, and the accuracy of the training set is 92.1%. The classification results of 340 test set samples by SVM algorithm are shown in Supplementary Figure S6B, 331 samples are accurately classified, only 9 samples are incorrectly classified, and the classification accuracy of the test set is 97.4%. It can be clearly seen that one sample of the third species was misclassified to the sixth species, five samples of the sixth species were misclassified to the first species, two samples of the sixth species were misclassified to the seventh species, and one sample of the seventh species was misclassified to the fifth species. Consequently, the algorithm has a low recognition rate for the sixth species (*Boletus bicolor*). Therefore, we need to find better identification methods.

### 3.4. Discrimination results of Alexnet model

**Figure 4:**
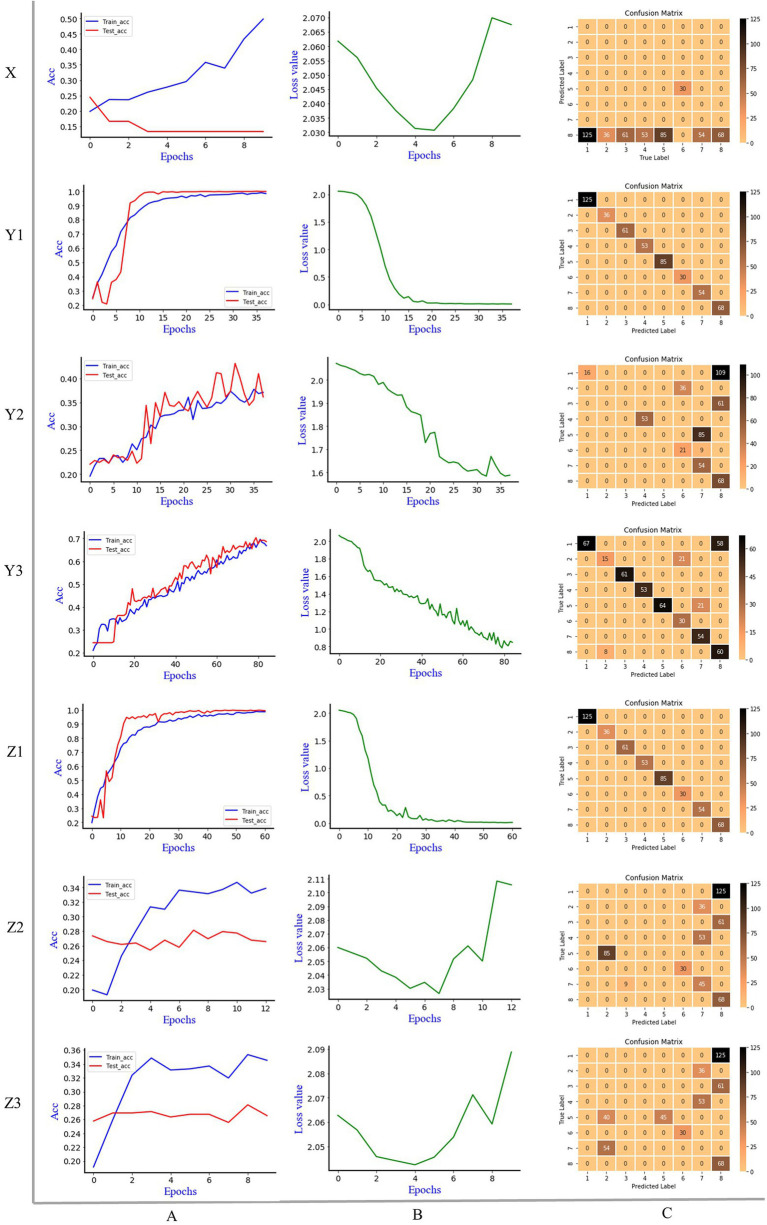
The discrimination results of Alexnet model, X: 1D MIR spectrum; Y1: synchronous 2DCOS; Y2: asynchronous 2DCOS; Y3: integrative 2DCOS; Z1: synchronous 3DCOS; Z2: asynchronous 3DCOS; Z3: integrative 3DCOS; A: accuracy curves; B: the cross-entropy cost function; C: the confusion matrix.

Figure 4 is the discrimination results of Alexnet model, where X is 1D MIR spectrum, Y is 2DCOS spectrum, Z is 3DCOS spectrum, A is the accuracy curves, B is the cross-entropy cost function, C is the confusion matrix. The number of samples in the training set was 1,195 (70%), and the number of samples in the test set was 512 (30%). In the Alexnet model, the initial learning rate is 0.01, the decay rate is 0.99, and the decay step is 3. To improve the convergence speed of the model, the Nesterov gradient descent method was used, and the learning rate was 0.001. Underfitting may occur if the number of epochs is too small. Conversely, if there are too many epochs, overfitting is likely to occur. Therefore, we use early stopping to select the number of epochs and stop training when the model’s performance on the test set does not increase. The parameter for early stopping is set as follows: monitor is the accuracy rate of the test set, min_delta is 0.001, patience is 5.

It can be seen that early stopping occurs on the 1D MIR spectrum dataset when the epoch is 10. The highest accuracy rate of the training set is 49.8%, while that of the test set is only 24.4%. The cross-entropy cost function decreases at first and then increases sharply, and the minimum loss value is 1.441, which is comparatively large. The confusion matrix showed that 68 samples of the eighth species (*Leccinum rugosiceps*) were correctly identified, and 444 samples of the other seven species were incorrectly identified. As a result, the model’s performance is poor in both accuracy and loss value. Early stopping occurs at the 38th epoch on the synchronous 2DCOS dataset. The accuracy rate is 98.9% on the training set and 100% on the test set. The minimum loss value is 0.038. All 512 samples in the confusion matrix were accurately classified, so the model performed well on this data set. For the asynchronous 2DCOS dataset, early stopping occurs at the 38th epoch. The accuracy rate is 37.6% on the training set and 43.2% on the test set. In addition, the minimum loss value is 1.618. The confusion matrix shows that 212 samples were correctly identified, and 300 samples were incorrectly identified. Therefore, the model of this data set is poor. The model stopped early at the 85th epoch on the Integrative 2DCOS dataset. The accuracy rate in the training set and test set is 69.6% and 79.3%, respectively. The minimum loss value is 0.817. In the confusion matrix, 100 samples were incorrectly identified and the rest were correctly identified. The performance of the model on this dataset is better than that of asynchronous 2DCOS, but markedly worse than that of synchronous 2DCOS. Consequently, synchronous 2DCOS has the best effect in the three 2DCOS datasets and can be used for the identification of boletes species.

On the synchronous 3DCOS dataset, the early stop occurs at the 61st epoch, and the accuracy of the training and test sets is 98.9% and 99.8%, respectively. The distribution of 512 samples in the test set on the confusion matrix is exactly correct. With the increase of epochs, the loss value gradually decreases, and the minimum value reaches 0.039, which is close to zero, indicating that the error of the model is small. It shows that the model performs well on this dataset and can be used for the identification of boletes species. For the asynchronous 3DCOS dataset, the model stops early at the 13th epoch. At this time, the accuracy is 34.7% on the training set and 28.1% on the test set, and the minimum loss value is 1.702, implying that the error of the model is large. The confusion matrix showed that only 143 samples of the sixth (*Boletus bicolor*), seventh (*Boletus speciosus*) and eighth (*Leccinum rugosiceps*) species were correctly identified. The accuracy of the training set and test set on integrative 3DCOS are 35.4% and 28.1%. Early stopping occurred at the 10th epoch, the minimum loss value was 1.652, and only 143 samples were correctly identified in the confusion matrix. These results show that the performance of the model on integrative 3DCOS and asynchronous 3DCOS datasets is similar, with low accuracy, high loss value and poor discrimination effect. Therefore, among the three 3DCOS datasets, Alexnet had the best performance in the synchronous 3DCOS dataset, which was suitable for the identification of boletes species.

### 3.5. Discrimination results of Resnet model

**Figure 5:**
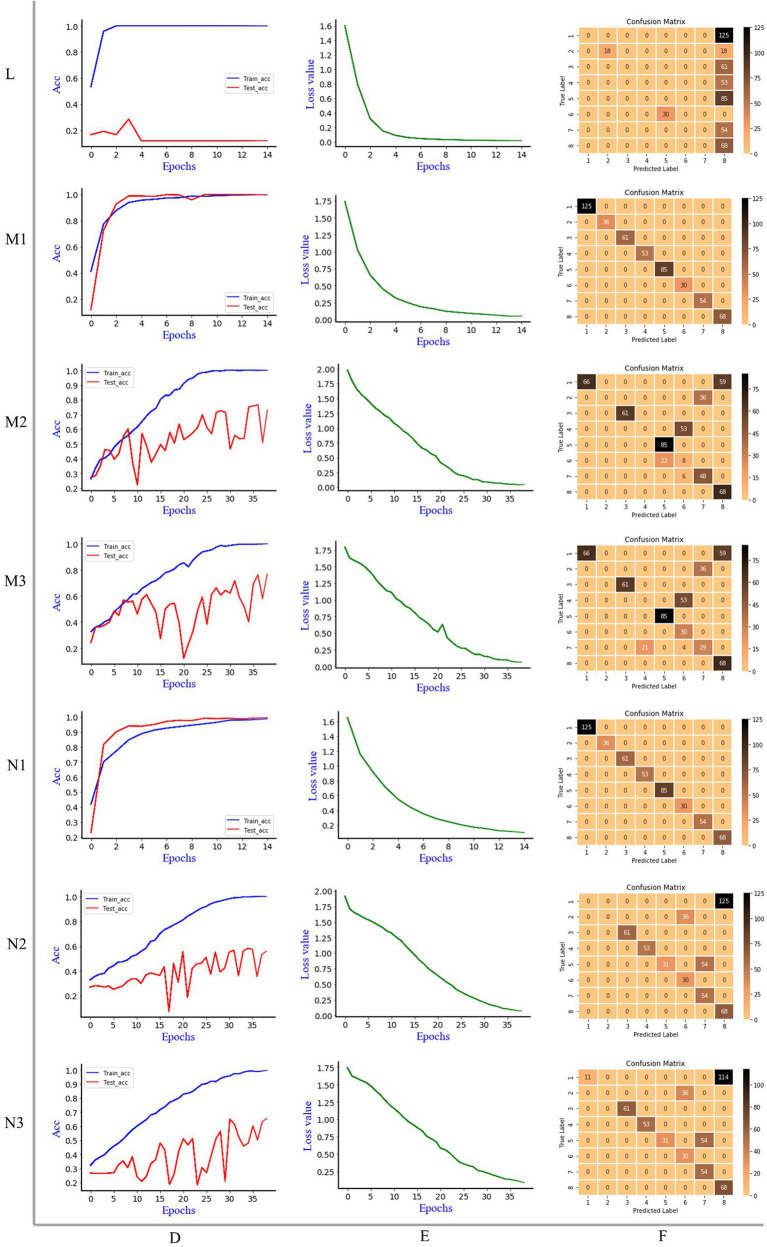
The discrimination results of Resnet model, L: 1D MIR spectrum; M1: synchronous 2DCOS; M2: asynchronous 2DCOS; M3: integrative 2DCOS; N1: synchronous 3DCOS; N2: asynchronous 3DCOS; N3: integrative 3DCOS; D: accuracy curves; E: the cross-entropy cost function; F: the confusion matrix.

Figure 5 is the discrimination results of Resnet model, where L is 1D MIR spectrum, M is 2DCOS spectrum, N is 3DCOS spectrum, D is the accuracy curves, E is the cross-entropy cost function, F is the confusion matrix. Same as the Alexnet model, the number of samples in the training set was 1,195 (70%), and the number of samples in the test set was 512 (30%). In the Resnet model, stochastic gradient descent (SGD) was used to modify the parameters of the optimization model to minimize the cross-entropy loss, L2 regularization was used to avoid overfitting, the weight decay factor was set to 0.0001, and the learning rate was 0.01.

When the epoch is 15, the accuracy of Resnet model on the training set is 100%, and the accuracy on the test set is 29%, and the minimum loss value is 0.014. Although the loss value is close to zero, the low accuracy of the test set leads to the poor effect of the model, which is not suitable for the identification of boletes species. This may be caused by overfitting due to the comparatively complex structure of the model due to the small amount of data. The confusion matrix demonstrated that only 18 samples of the second species (*Sutorius magnificus*) and 68 samples of the eighth species (*Leccinum rugosiceps*) were correctly identified, while the rest were incorrectly identified. Figure 5 shows that the Resnet model performs better on the synchronous 2DCOS dataset. When the epoch is 15, the accuracy of the training set and test set is 100%, and 512 samples are correctly identified in the confusion matrix. At this time, the loss value is 0.052, which is close to zero. This indicates that the error of the model is small. Therefore, the synchronous 2DCOS model has high accuracy, small loss value, and strong generalization ability. The model can be used for the identification of boletes species. When the epoch is 39, the accuracy of the Resnet model on the training set and test set is 100% and 77%, respectively, on the asynchronous 2DCOS dataset, and the loss value is 0.041. The confusion matrix indicated that 313 samples were correctly identified and the remaining 189 samples were incorrectly identified. Although the loss value is small, the accuracy of the test set is low and the generalization ability is poor, which leads to the unavailability of the model. Similarly, when the epoch is 39, the accuracy of the Resnet model on the integrative 2DCOS dataset is 100% and 77% for the training and testing sets, and the loss value is 0.064. The confusion matrix revealed that 339 samples were correctly identified and the remaining 173 samples were incorrectly identified. Therefore, the generalization ability of this model is poor and it is not available. Therefore, in the three datasets of 2DCOS, Resnet performs perfectly on the synchronous 2DCOS dataset, while the test set accuracy on the other two datasets is relatively low.

Compared with synchronous 2DCOS, the accuracy of the Resnet model in the training and testing sets on the synchronous 3DCOS dataset is also 100%. Although the loss value is 0.1, which is higher than that of the synchronous 2DCOS dataset, its epoch is only 15, which saves the computational complexity and time cost. All test samples are correctly identified in the confusion matrix. As a result, the Resnet model shows different advantages on synchronous 2DCOS and synchronous 3DCOS datasets, which are both perfect models. When the epoch is 39, the loss value of the Resnet model on the asynchronous 3DCOS dataset is 0.07. The accuracy was 100% on the training set and 58% on the test set. In the confusion matrix, 297 samples were accurately identified and 215 samples were incorrectly identified. In the case of the same epoch value, the loss value of the Resnet model on the integrative 3DCOS dataset is 0.09. The accuracy on the training set was 100%, while the accuracy on the test set was 65%. In the confusion matrix, 308 samples were correctly identified and 204 samples were incorrectly identified. Therefore, the effect of Resnet model on integrative 3DCOS and asynchronous 3DCOS datasets is similar with relatively low accuracy, while the effect of Resnet model on synchronous 3DCOS datasets is the best, which can be applied to actual species discrimination.

In addition, by analyzing the confusion matrix, we found that the Alexnet and Resnet models performed best in identifying the eighth species (*Leccinum rugosiceps*) in all data sets, with almost no misjudgment. This may be related to the chemical characteristics, molecular structure, phenological characteristics and growing environment of this species, which lead to the obvious characteristics of its spectral image compared with other species and make it easier to identify.

### 3.6. The comparison of model results

Table 1 shows the results of species identification of boletes species using various models. It can be seen that the Resnet model has the best effect on the synchronous 2DCOS dataset. The accuracy of the training and test sets is 100% which shows that the model has high accuracy. The loss value is close to zero, which indicates that the error of the model is small. The epoch value is only 15 and the time cost is 41.5 min, which proves that the model works quickly. The external verification is all correct, which indicates that the model has high reliability and strong generalization ability. In addition, the deep learning model processes spectral images directly without complex data parsing, which is simple and convenient. The Resnet model also performs well on the synchronous 3DCOS dataset. The accuracy of the training and testing sets is 100%, and the epoch value is 15. Nevertheless, the loss value is 0.1 and the time cost is 66.6 min. It takes more time to achieve the same effect as the synchronous 2DCOS dataset, and the loss value also increases. The accuracy of the Alexnet model on the synchronous 2DCOS dataset is 98.9% for the training set and 100% for the test set, and the epoch value is 38. However, the loss value is 0.038, and the time cost is 66.3 min. The training effect of the model on the synchronous 3DCOS dataset is similar to that on the synchronous 2DCOS dataset. The accuracy of the training set is 98.9%, the accuracy of the test set is 99.8%, and the loss value is 0.039, but the epoch expands to 61. The time cost increases to 152.1 min, more than twice as much as on the Synchronous 2DCOS dataset. The above four models can be used for the identification of boletes species. Among them, the Resnet model has the highest accuracy, the best effect, the smallest error and the least time cost on the synchronous 2DCOS dataset. In this case, the sensitivity of the model is 100%, which indicates that the model is relatively stable. As shown in Supplementary Figure S1, four boletes species (*Sutorius magnificus, Boletus speciosus, Boletus bainiugan, Boletus bicolor*) have similar appearance, which are difficult to distinguish in morphology. However, the Resnet model on the synchronous 2DCOS dataset can be used to identify them accurately, and the external verification accuracy is 100%, which can be inferred that the sensitivity of the model is very high.

**Table 1:** The comparison of model results.

Methods	Dimensions of images	Image types	Epoch	Loss value	Train set accuracy	Test set accuracy	Time costs (minutes)
SVM	/	/	/	/	92.10%	97.35%	1,092
Alexnet	1D	The MIR spectrum	10	2.032	49.80%	24.40%	21.2
	2D	synchronous	38	0.038	98.90%	100.00%	66.3
		asynchronous	38	1.618	37.60%	43.20%	68.2
		integrative	85	0.817	69.60%	79.30%	183.1
	3D	synchronous	61	0.039	98.90%	99.80%	152.1
		asynchronous	13	1.702	34.70%	28.10%	23.2
		integrative	10	1.652	35.40%	28.10%	20.3
Resnet	1D	The MIR spectrum	15	0.014	100%	29%	43.1
	2D	synchronous	15	0.052	100%	100%	41.5
		asynchronous	39	0.041	100%	77%	178.6
		integrative	39	0.064	100%	77%	125.2
	3D	synchronous	15	0.1	100%	100%	66.6
		asynchronous	39	0.07	100%	58%	157.3
		integrative	39	0.09	100%	65%	152.8

The SVM model does not process the spectral images, but trains the original spectral data directly. Although the accuracy of the training set is 92.1% and the test set is 97.35%, it is much higher than the accuracy of Resnet and Alexnet models on asynchronous 2DCOS, Integrative 2DCOS, asynchronous 3DCOS and Integrative 3DCOS datasets. However, under the same hardware conditions, the training time is as high as 1,092 min, so the time cost of this model is too high, and it is not recommended to be used. In general, Resnet model is better than Alexnet and SVM model.

**Figure 6:**
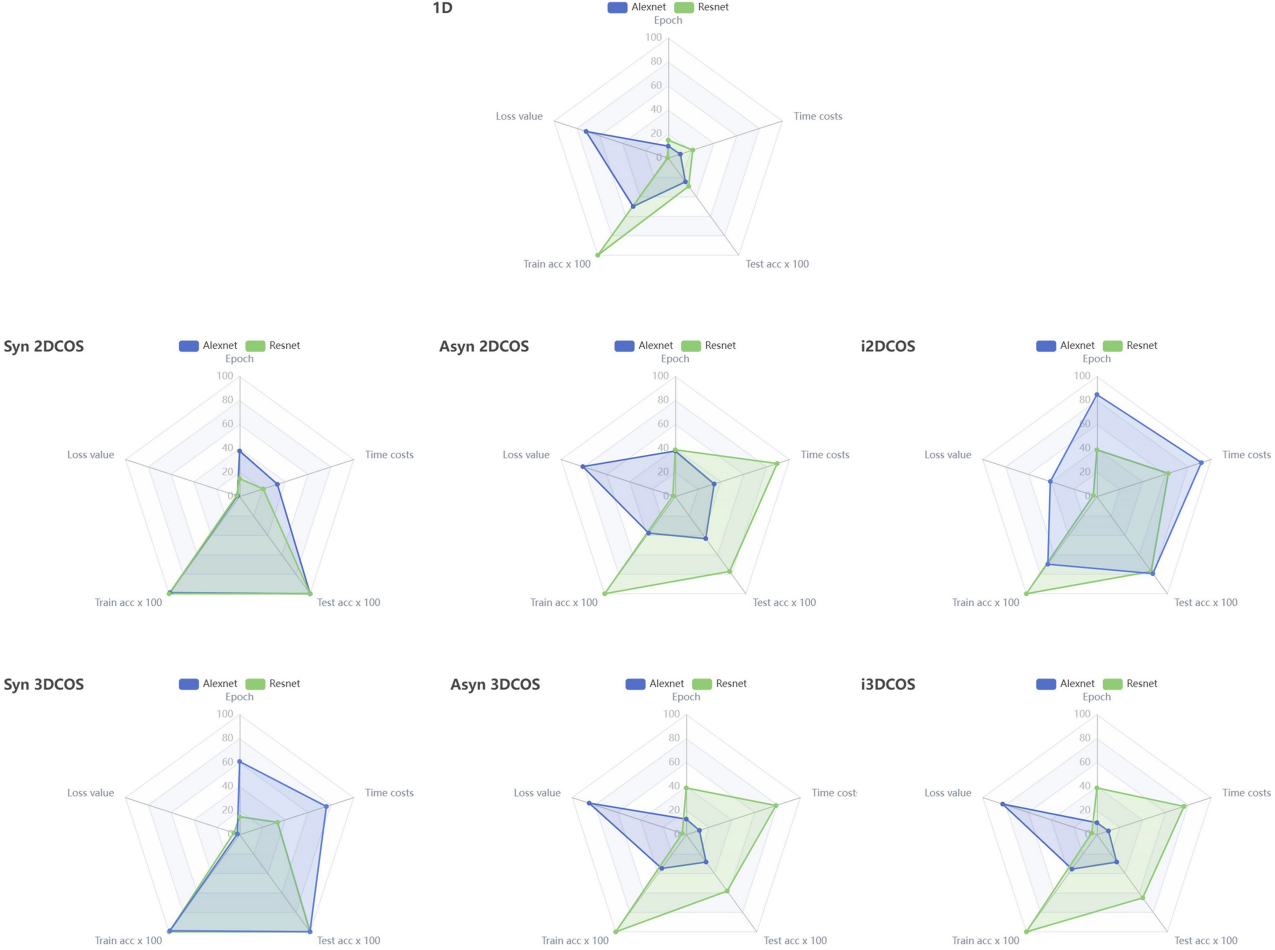
Parameter comparison between Alexnet and Resnet models in all data sets, 1D: The original MIR spectrum; Syn: synchronous; Asyn: asynchronous; i2DCOS: integrative 2DCOS; i3DCOS:integrative 3DCOS.

Figure 6 compares the parameters of Resnet and Alexnet models on different data sets through radar charts. We can see that these two models perform best on synchronous spectral images (including 2DCOS and 3DCOS), where Resnet has smaller epochs and lower time complexity compared to Alexnet. The two models performed the worst on the original MIR spectrum, and their test set had the lowest accuracy. Although the model has a high accuracy in the training set on the other four data sets, the accuracy in the test set is not high, and Alexnet has a high loss value, and the time cost of Resnet is relatively high. In summary, synchronous 2DCOS and synchronous 3DCOS datasets perform better than other datasets. This may be because the synchronous spectral image contains more auto-peaks and cross-peaks, which leads to its obvious features and makes it easier to adopt the deep learning method for image recognition. However, the original MIR Spectrum image is a simple curve, which contains less feature information and its feature peaks overlap, so its recognition effect is the worst.

### 3.7. Visualization analysis

**Figure 7:**
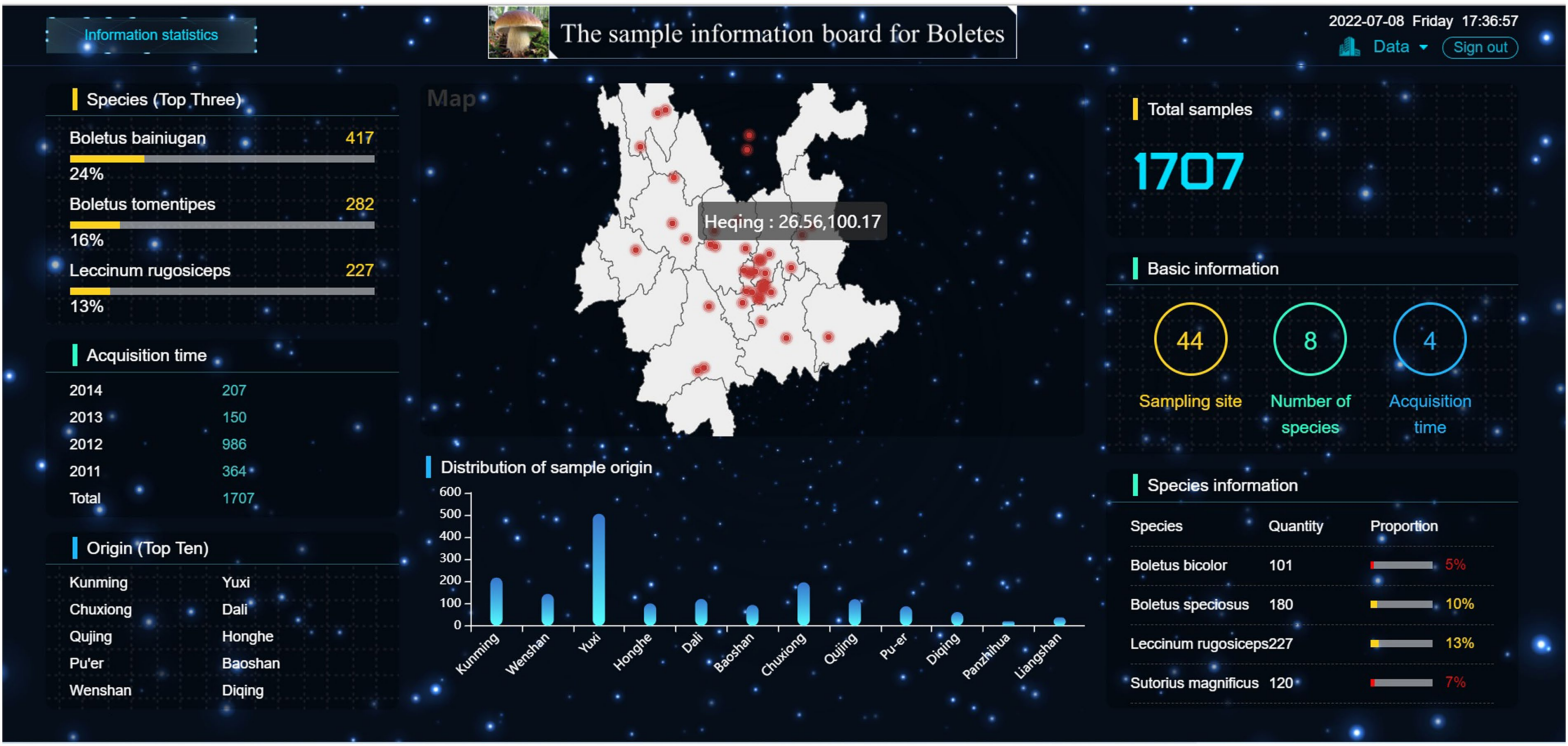
Visual large-screen.

Figure 7 displays the detailed information of the samples in this research. The right side of the large-screen shows that the sample size of this study is 1707, including 44 sampling sites, eight boltetes species and 4 sample collection years. The map module in the middle shows the sampling sites and their latitude and longitude. It can be seen that the sampling sites practically cover the whole Yunnan Province, with the most dense distribution in Central Yunnan, followed by the Northwest Yunnan, and fewer sampling sites in Northeast Yunnan. In addition, it can be seen that there are two sampling sites in southern Sichuan Province. The main reason is that boletes is abundant in Central Yunnan and Northwest Yunnan due to the influence of climate and environmental conditions. This is also proved by the Origin (Top 10) and distribution of sample origin modules. From the distribution of sample origin module, it can be seen that Yuxi has the largest number of samples, more than 500, while Liangshan, Panzhihua and Diqing have the smallest number of samples, among which Panzhihua has only 20 samples. In the Species (Top Three) module on the left, it can be seen that *Boletus Bainiugan* has the largest number, with 417 samples, accounting for 24% of the total sample size, followed by *Boletus Tomentipes* and *Leccinum Rugosiceps*. They accounted for 16 and 13% of the total sample size, which implied that Yunnan was rich in these types of boletus. From the perspective of acquisition time, the largest sample size was collected in 2012, which may be due to the abundant rainfall in Yunnan from July to September 2012, which promoted the high yield of boletes.

## 4. Conclusion

In this paper, based on deep learning, a method using 2DCOS and 3DCOS spectral image processing and recognition technology is proposed to identify the species of boletes. Among them, 3DCOS was proposed for the first time. Experimental results show that the method is rapid, accurate and effective. In our study, 15 models were established through three algorithms and eight datasets. Although the accuracy rate of SVM algorithm was higher than 90%, the time cost of is too high, and it needed to run for more than 18 h on our hardware. The remaining 14 models were based on deep learning method, and the results show that the Resnet algorithm is the best model in the synchronous2DCOS dataset with 100% accuracy, small loss value and low time complexity. In addition, the detailed information of all samples is displayed visually on a large visual screen, and the correlation analysis is carried out to obtain the relevant conclusion of the sample distribution. This research overcomes previous problems such as single algorithm, lack of data sets and less choice caused by fewer models. In summary, the method recommended in this paper is effective and reliable, and this method can be applied to other discrimination fields in future research.

## Data availability statement

The original contributions presented in the study are included in the article/supplementary material, further inquiries can be directed to the corresponding authors.

## Author contributions

J-QL: conceptualization. Y-ZW: ethodology and writing—original draft, formal analysis, data curation, and project administration. HL: resources and supervision. All authors contributed to the article and approved the submitted version.

## Funding

This work was supposed by the National Natural Science Foundation of China (Grant number: 32160735).

## Conflict of interest

The authors declare that the research was conducted in the absence of any commercial or financial relationships that could be construed as a potential conflict of interest.

## Publisher’s note

All claims expressed in this article are solely those of the authors and do not necessarily represent those of their affiliated organizations, or those of the publisher, the editors and the reviewers. Any product that may be evaluated in this article, or claim that may be made by its manufacturer, is not guaranteed or endorsed by the publisher.

## Footnotes

^1^ http://www.yncoop.com/sxjs/zsjs/08196173330559241148
