# Supplementary material for: Application of spectral image processing with different dimensions combined with large-screen visualization in the identification of boletes species
Source: Front Microbiol. 2023 Jan 12;13:1036527. doi: 10.3389/fmicb.2022.1036527 (PMC9877520; doi:10.3389/fmicb.2022.1036527)
Supplement: Supplementary file 1 [file Data_Sheet_1.docx]

Supplementary Material

# Supplementary Figures and Tables

## 1.1 Supplementary Figures

Fig. s1 The appearances of eight boletes species

**Fig. s2** The Alexnet model

**Fig. s3** The Resnet model

**Fig. s4** The identification strategy of boletes species

Fig. s5 The layout and proportion of large-screen panels

Fig. s6 The discrimination results of SVM model

## 1.2 Supplementary Tables

**Table s1** Information of the samples

**Table s2** The detailed data set partition

**Table s3** Peak assignments on the FT-MIR spectra of boletus


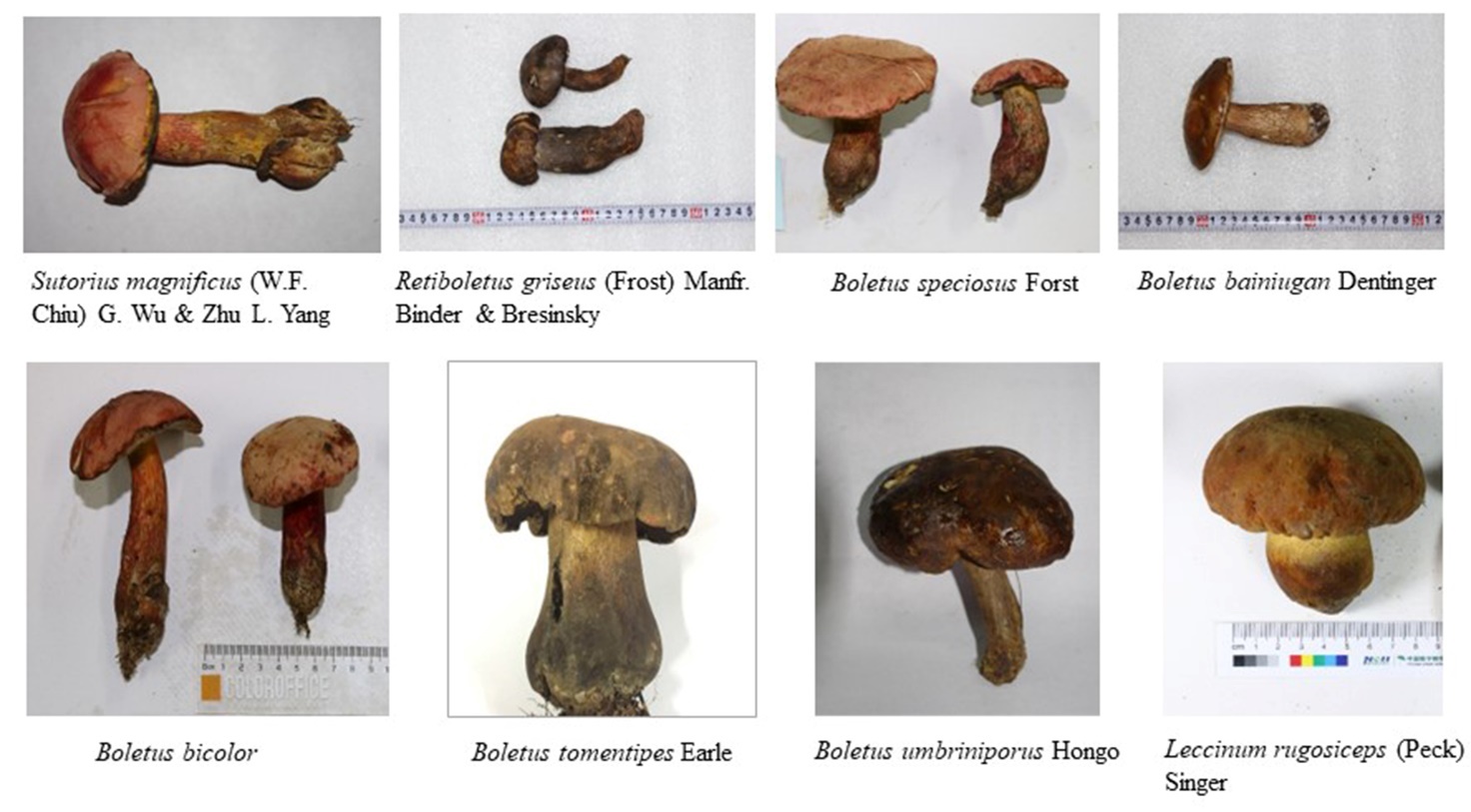


Fig. s1 The appearances of eight boletes species


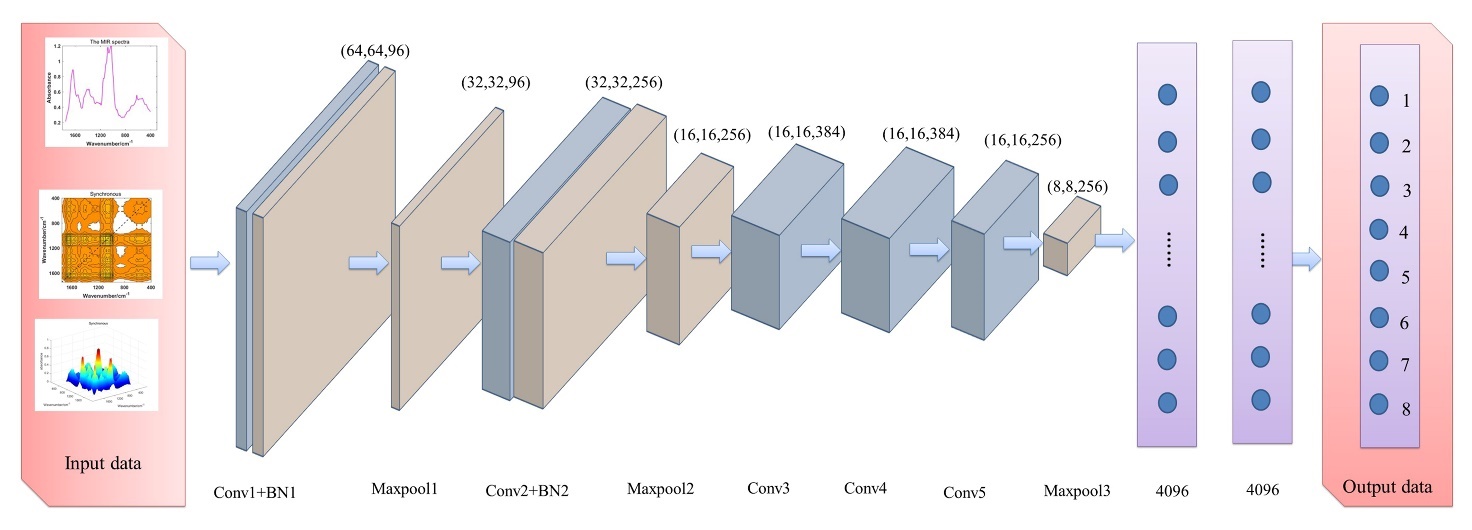


**Fig. s2** The Alexnet model


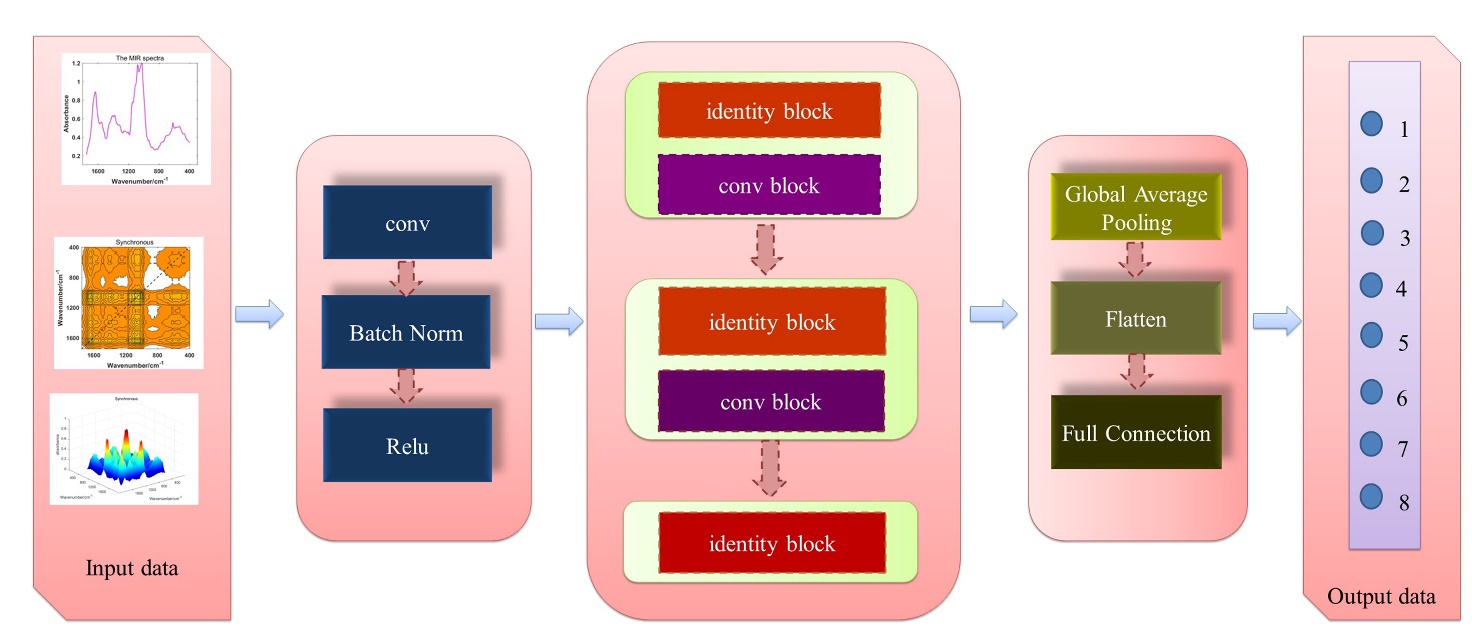


**Fig. s3** The Resnet model


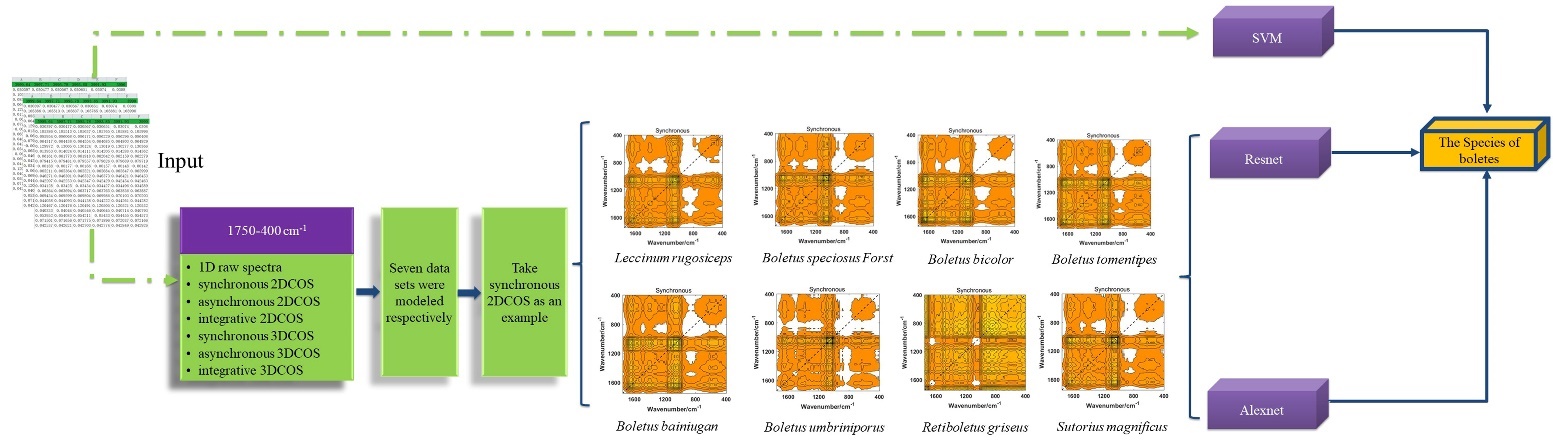


**Fig. s4** The identification strategy of boletes species


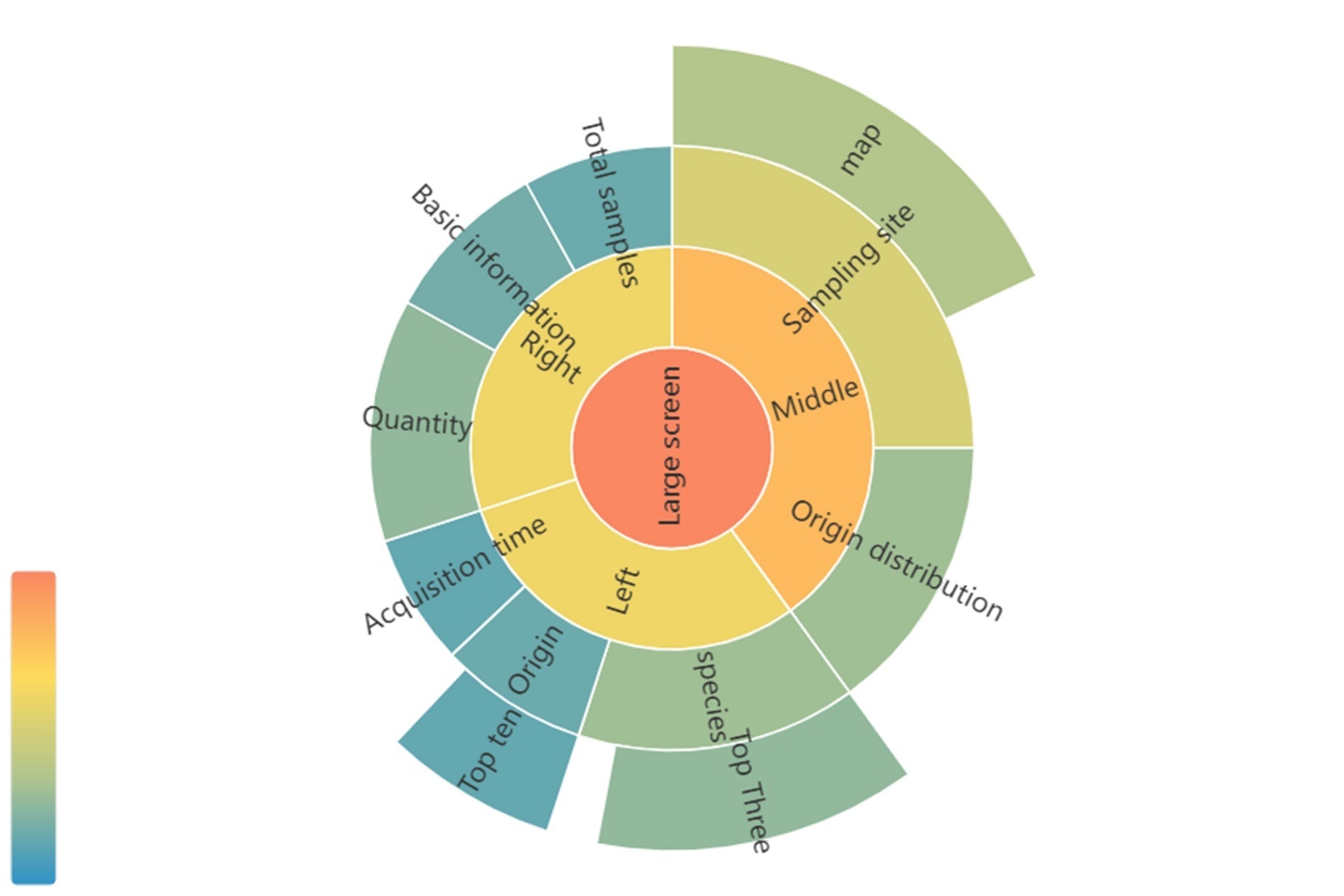


Fig. s5 The layout and proportion of large-screen panels


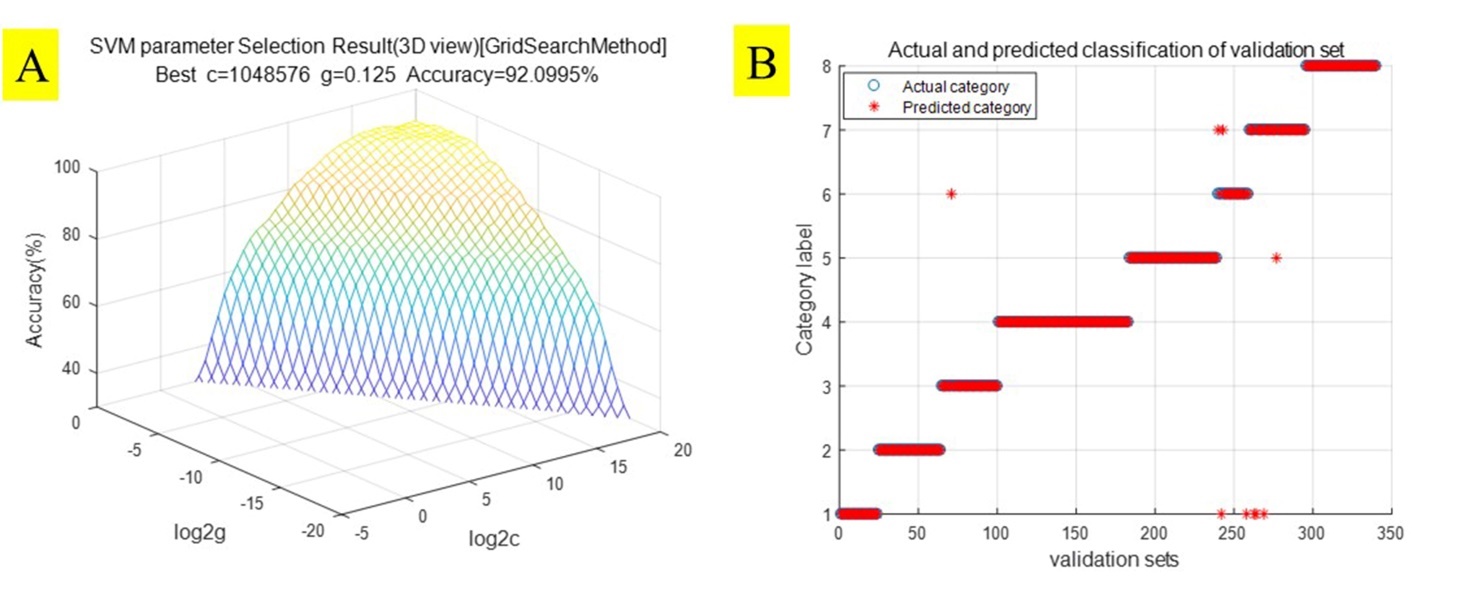


Fig. s6 The discrimination results of SVM model

**Table s1** Information of the samples

| **Class** | **Name** | **Sampling site** | **Number of fruiting body** | **Latitude ( ◦ N)** | **Longitude ( ◦ E)** |
| --- | --- | --- | --- | --- | --- |
| 1 | *Sutorius magnificus (W.F. Chiu) G. Wu & Zhu L. Yang* | Jiangchuan,Yuxi | 20 | 24.28 | 102.75 |
|  |  | Yimentongchang,Yxi | 20 | 24.71 | 102.03 |
|  |  | Gejiu,Honghe | 14 | 23.37 | 103.15 |
|  |  | Yimenjiangkou,Yuxi | 14 | 24.66 | 102.22 |
|  |  | Midu,Dali | 18 | 25.34 | 100.49 |
|  |  | Simao,Pu'er | 20 | 22.78 | 100.97 |
|  |  | Dayingjie,Yuxi | 14 | 24.33 | 102.49 |
| 2 | *Retiboletus griseus (Frost) Manfr. Binder & Bresinsky* | Eshan,Yuxi | 20 | 24.16 | 102.4 |
|  |  | Wuhuaqu,Kunming | 16 | 25.04 | 102.70 |
|  |  | Longyangqu,Baoshan | 20 | 25.12 | 99.16 |
|  |  | Jiangchuan,Yuxi | 19 | 24.28 | 102.75 |
|  |  | Midu,Dali | 19 | 25.34 | 100.49 |
|  |  | Jinning,Kunming | 14 | 24.66 | 102.59 |
|  |  | Malong,Qujing | 14 | 25.42 | 103.57 |
|  |  | Anning,Kunming | 14 | 24.91 | 102.47 |
|  |  | Shilin,Kunming | 20 | 24.77 | 103.28 |
|  |  | Lufeng,Chuxiong | 20 | 25.15 | 102.07 |
|  |  | Zhenyuan,Pu'er | 12 | 24.00 | 101.10 |
|  |  | Dayingjie,Yuxi | 14 | 24.33 | 102.49 |
| 3 | *Boletus umbriniporus Hongo* | Yimen,Yuxi | 40 | 24.67 | 102.16 |
|  |  | Longyangqu,Baoshan | 20 | 25.12 | 99.16 |
|  |  | Midu,Dali | 16 | 25.34 | 100.49 |
|  |  | Shiping,Honghe | 18 | 23.70 | 102.49 |
|  |  | Yuanmou,Chuxiong | 20 | 25.70 | 101.87 |
|  |  | Huangcaoba,Yuxi | 16 | 24.46 | 102.61 |
|  |  | Gejiu,Honghe | 20 | 23.37 | 103.15 |
|  |  | Hongtaqu,Yuxi | 2 | 24.34 | 102.54 |
|  |  | Zezhou,Qujing | 10 | 25.60 | 103.82 |
|  |  | Nanhua,Chuxiong | 16 | 25.19 | 101.27 |
| 4 | *Boletus bainiugan Dentinger* | Yimen,Yuxi | 76 | 24.67 | 102.16 |
|  |  | Nanhua,Chuxiong | 36 | 25.19 | 101.27 |
|  |  | Nanhuashaqiao,Chuxiong | 16 | 25.23 | 101.14 |
|  |  | Pudacuo,Diqing | 20 | 27.90 | 99.95 |
|  |  | Midu,Dali | 20 | 25.34 | 100.49 |
|  |  | Weixi,Diqing | 14 | 27.17 | 99.28 |
|  |  | Longyangqu,Baoshan | 20 | 25.12 | 99.16 |
|  |  | Anningwenshui,Kunming | 20 | 24.92 | 102.41 |
|  |  | Anningfengyi,Kunming | 20 | 24.69 | 102.33 |
|  |  | Heqing,Dali | 14 | 26.56 | 100.17 |
|  |  | Dongshan,Wenshan | 14 | 23.39 | 104.27 |
|  |  | Anningbajie,kunming | 16 | 24.92 | 102.48 |
|  |  | Shilin,Kunming | 20 | 24.77 | 103.28 |
|  |  | Malong,Qujing | 30 | 25.42 | 103.57 |
|  |  | Zezhou,Qujing | 8 | 25.60 | 103.82 |
|  |  | Dayingjie,Yuxi | 32 | 24.33 | 102.49 |
|  |  | Zhenyuan,Pu'er | 7 | 24.00 | 101.10 |
|  |  | Xinping,Yuxi | 14 | 24.07 | 101.99 |
|  |  | Jiulongchi,Yuxi | 20 | 24.42 | 102.52 |
| 5 | *Boletus tomentipes Earle* | Eshanfuliangpeng,Yuxi | 18 | 24.30 | 102.09 |
|  |  | Eshanxiaojie,Yuxi | 18 | 24.15 | 102.45 |
|  |  | Yaoan,Chuxiong | 14 | 25.50 | 101.24 |
|  |  | Nanhua,Chuxiong | 14 | 25.19 | 101.27 |
|  |  | Nanbanghe,Pu'er | 21 | 22.72 | 100.80 |
|  |  | Zezhou,Qujing | 14 | 25.60 | 103.82 |
|  |  | Gejiu,Honghe | 14 | 23.37 | 103.15 |
|  |  | Shangri-la,Diqing | 17 | 27.84 | 99.74 |
|  |  | Yimen,Yuxi | 20 | 24.67 | 102.16 |
|  |  | Eshanchahe,Yuxi | 14 | 24.28 | 102.24 |
|  |  | Shiping,Honghe | 16 | 23.70 | 102.49 |
|  |  | Dechang,Liangshan | 18 | 27.40 | 102.17 |
|  |  | Heqing,Dali | 14 | 26.56 | 100.17 |
|  |  | Miyi,Panzhihua | 20 | 27.11 | 102.11 |
|  |  | Xinping,Yuxi | 20 | 24.07 | 101.99 |
|  |  | Eshanshuangjiang,Yuxi | 12 | 24.16 | 102.41 |
|  |  | Dayingjie,Yuxi | 18 | 24.33 | 102.49 |
| 6 | *Boletus bicolor* | Nanhua,Chuxiong | 13 | 25.19 | 101.27 |
|  |  | Cangshan,Dali | 14 | 25.65 | 100.13 |
|  |  | Zezhou,Qujing | 14 | 25.60 | 103.82 |
|  |  | Jiangchuan,Yuxi | 20 | 24.28 | 102.75 |
|  |  | Dayingjie,Yuxi | 32 | 24.33 | 102.49 |
|  |  | Zhenyuan,Pu'er | 8 | 24.00 | 101.10 |
| 7 | *Boletus speciosus Forst* | Nanhua,Chuxiong | 14 | 25.19 | 101.27 |
|  |  | Yimen,Yuxi | 20 | 24.67 | 102.16 |
|  |  | Anning,Kunming | 40 | 24.91 | 102.47 |
|  |  | Longyangqu,Baoshan | 20 | 25.12 | 99.16 |
|  |  | Yuanmou,Chuxiong | 20 | 25.70 | 101.87 |
|  |  | Zezhou,Qujing | 10 | 25.60 | 103.82 |
|  |  | Dayingjie,Yuxi | 16 | 24.33 | 102.49 |
|  |  | Eshan,Yuxi | 20 | 24.16 | 102.4 |
|  |  | Beicheng,Yuxi | 20 | 24.42 | 102.55 |
| 8 | *Leccinum rugosiceps (Peck) Singer* | Wuhuaqu,Kunming | 16 | 25.04 | 102.70 |
|  |  | Zezhou,Qujing | 20 | 25.60 | 103.82 |
|  |  | Yimen,Yuxi | 34 | 24.67 | 102.16 |
|  |  | Simao,Pu'er | 20 | 22.78 | 100.97 |
|  |  | Cangshan,Dali | 20 | 25.65 | 100.13 |
|  |  | Shiping,Honghe | 19 | 23.70 | 102.49 |
|  |  | Midu,Dali | 20 | 25.34 | 100.49 |
|  |  | Anning,Kunming | 20 | 24.91 | 102.47 |
|  |  | Weixi,Diqing | 14 | 27.17 | 99.28 |
|  |  | Shilin,Kunming | 16 | 24.77 | 103.28 |
|  |  | Longyangqu,Baoshan | 14 | 25.12 | 99.16 |
|  |  | Yuanmou,Chuxiong | 14 | 25.70 | 101.87 |
|  |  | **Total Number** | **1707** |  |  |

**Table s2** The detailed data set partition

| **Class** | **Species** | **The total number of samples** | **SVM train set** | **SVM test set** | **Resnet/Alexnet train set** | **Resnet/Alexnet test set** |
| --- | --- | --- | --- | --- | --- | --- |
|  |  |  | **80%** | **20%** | **70%** | **30%** |
| 1 | *Boletus bainiugan* Dentinger | 417 | 334 | 83 | 292 | 125 |
| 2 | *Sutorius magnificus* (W.F. Chiu) G. Wu & Zhu L. Yang | 120 | 96 | 24 | 84 | 36 |
| 3 | *Retiboletus griseus* (Frost) Manfr. Binder & Bresinsky | 202 | 162 | 40 | 141 | 61 |
| 4 | *Boletus umbriniporus* Hongo | 178 | 142 | 36 | 125 | 53 |
| 5 | *Boletus tomentipes* Earle | 282 | 226 | 56 | 197 | 85 |
| 6 | *Boletus bicolor* | 101 | 81 | 20 | 71 | 30 |
| 7 | *Boletus speciosus Forst* | 180 | 144 | 36 | 126 | 54 |
| 8 | *Leccinum rugosiceps (Peck) Singer* | 227 | 182 | 45 | 159 | 68 |

**Table s3** Peak assignments on the FT-MIR spectra of boletus

| **Band (cm-1)** | **Wavenumber (cm-1)** | **Assignment** | **Component** | **Ref.** |
| --- | --- | --- | --- | --- |
| 3600-3200 | 3342 | O-H stretching | water molecule | (Hirri et al., 2016; Wang, 2020) |
| 3000-2850 | 2928 | stretch of methylene group of lipid | fatty acid | (Mohacek-Grosev et al., 2001; Zhao et al., 2015; He, 2019) |
|  | 2855 | pyranose ring |  |  |
| 1700-1000 | 1700-1650 | organic material, C-C stretching,C-O-H、 C-H and CH2 bending | proteins | (Nie et al., 2007; Yang et al., 2014; Zhang et al., 2018; Chen et al., 2021; Dong et al., b) |
|  | 1650-1500 |  | amide І and amide П |  |
|  | 1450-1200 |  | proteins, fatty acids and polysaccharides |  |
|  | 1000-1200 |  | carbohydrate |  |
|  | 1032 and 1080 |  | chitin |  |
| 900-400 | 900-800 | The fingerprint region where many peaks are unidentified | glucan and mannan | (Qi et al., 2018; Chen et al., 2021) |
